# Development of an undergraduate otorhinolaryngology simulation education and human factors module: its impact on students’ attitudes and perceptions

**DOI:** 10.1017/S0022215125103009

**Published:** 2025-11

**Authors:** Niall James McInerney, Gerard P. Sexton, Adam Roche, Martin Donnelly, Liam Skinner

**Affiliations:** 1 Royal College of Surgeons in Ireland, Dublin, Ireland; 2 Department of Otorhinolaryngology – Head & Neck Surgery, University Hospital Waterford, Waterford, Ireland

**Keywords:** otolaryngology, simulation training

## Abstract

**Objectives:**

To evaluate the impact of a two-week otolaryngology rotation incorporating entrustable professional activities, human factors and simulation on medical students’ knowledge, perceptions and career aspirations.

**Methods:**

The curriculum included six small-group sessions on compassion, communication, resilience, teamwork and professionalism, and three simulations: suturing, flexible nasendoscopy and grommet insertion. These were delivered alongside standard teaching. Pre- and post-rotation questionnaires assessed otolaryngology knowledge, career interest, surgical confidence and attitudes toward simulation and human factors.

**Results:**

While students’ interest in surgical careers remained unchanged, they reported improved comfort with otolaryngology knowledge, operating theatre environments and recognition of non-technical skills. Perceptions of simulation and essential surgeon qualities significantly improved.

**Conclusion:**

Integrating entrustable professional activities, human factors education and simulation into short surgical rotations enhances both technical and non-technical skills. This approach may help address challenges in attracting students to surgery by enriching their educational experience and building confidence.

## Introduction

Undergraduate medical education is based on traditional structures, with students initially acquiring knowledge from didactic lectures in the early years of medical school, and then this knowledge is applied in the later years of clinical placement.^1^ Recently, the benefits of the addition of simulation to the curriculum have been documented.^2^ Simulation programmes are most frequently used in the core medical specialities, often covering emergency scenarios. Subspeciality education is generally completed over a shorter period, and as such there is often not adequate time to incorporate simulation training. As a result, some students may not get enough exposure in some subspecialities to spark their interest for their future careers. Furthermore, their perception of surgical specialties can be biased, with many believing that burnout and a poor work–life balance are typical of some specialities.^3^

Using a novel education initiative, we sought to educate our students not only on key otolaryngology topics, but also on the technical skills and non-technical attributes required for surgical training. Our primary aim was to educate students on common otolaryngology topics. These topics were covered in the core curriculum, but with the addition of simulation training, key messages were reinforced. It is accepted that the addition of simulation training to didactic learning can lead to greater levels of understanding and enhanced retention.^2^ Secondly, our simulation and human factors module aimed to prepare students for working life and the hospital environment. Students on their early clinical placements can feel intimidated and overwhelmed when they start the placement, which can have a negative impact on their education.^3^ Our module aimed to ensure students felt more prepared to deal with everyday scenarios and had the capabilities to deal with difficult situations in a calm and measured manner.

The Royal College of Surgeons in Ireland has introduced mandatory human factors and patient safety modules for surgeons in training, and lessons from teaching and participating in these courses were implemented in this module.^4^ We endeavoured to implement five key concepts to ensure the highest standards in healthcare delivery and education were achieved. These concepts were transparency, care integration, patient engagement, restoration of joy and meaning in work, and medical education reform.^5^

Finally, a secondary endpoint of our study was to foster interest in a career in otolaryngology. Attracting medical students and young doctors into a career in surgery is a multilayered, challenging issue. The demanding nature of surgical training, with its long hours and rigorous training pathway, is just one deterrent.^6^ Balancing work–life considerations and concerns about burnout and evolving public perceptions and demands are other factors that come into play.^6^ We aimed to educate students on the technical and non-technical skills required in otolaryngology so that they could experience the challenges faced when executing these skills.

## Methods

### Study participants

The focus of this study was to assess the attitudes and usefulness of a new simulation programme for senior undergraduate students commencing their clinical learning in otolaryngology following two or three years of preclinical education for graduate entry and direct undergraduate medicine students, respectively. Students at this stage are divided into groups and assigned to clinical attachments in different medical specialities for two weeks. No academic parameters, such as grade point average or prior examination results, are taken into account for group assignment. All students share a common lecture series in otolaryngology prior commencing our module, which includes common pathologies, history taking and physical examinations.

### Simulation facility and programme

**Figure 1.**
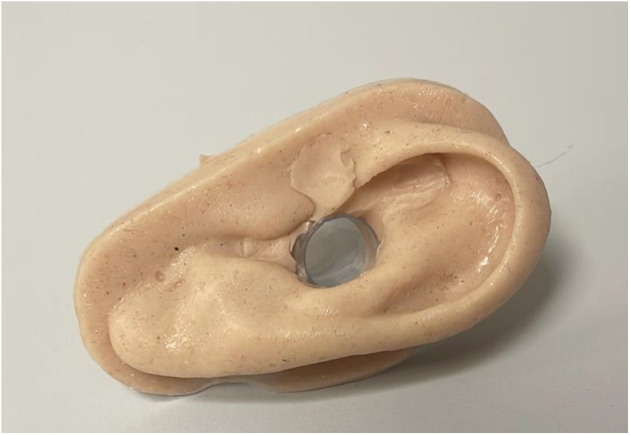
Tympanostomy tube simulation model.

**Figure 2.**
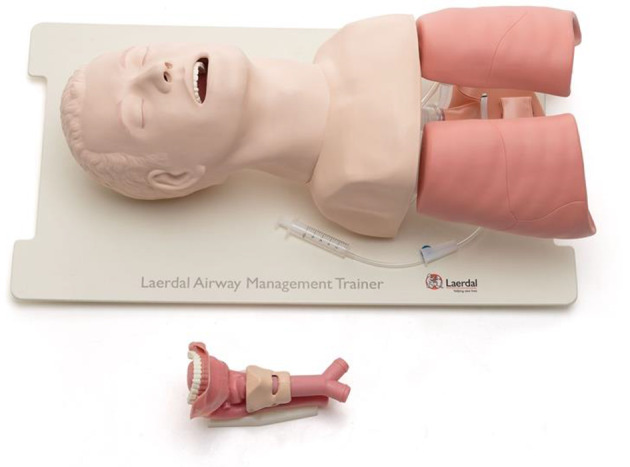
Laerdal® Airway Management Trainer.

Simulation took place in a clinical procedure room, with all equipment used similar to everyday clinical equipment. The Laerdal® Airway Management Trainer (Figure 2) and Ambu® aScope™ were used for flexible nasendoscopy. A novel ear manikin (Figure 1) was developed for tympanostomy tube insertion, which was used along with an operating microscope (Karl Storz, Tuttlingen, Germany).

Our study focused on enhancing medical students’ education in otolaryngology through a two-week rotation using entrustable professional activities. This approach was complemented by six discussions on human factor topics emphasising compassionate care, effective communication, resilience building, teamwork and professionalism.

### Study design

To assess the usefulness of these simulation sessions, we assessed student attitudes by questionnaire prior to commencing the module and on the concluding day of the module. The study was conducted in accordance with the ethical principles outlined in the Declaration of Helsinki and adhered to all applicable institutional and national research ethics guidelines.

### Attitude questionnaire

Pre- and post-attachment questionnaires were administered, assessing students’ career aspirations, prior simulation training experience, perceived qualities for a surgeon, objectives for the ENT rotation, comfort levels with otolaryngology knowledge, comfort in an operating theatre setting, the importance of simulation in surgical training and the significance of human factors in their training.

### Statistical analysis

The collected data were interrogated for normality (Shapiro–Wilk test, *p* < 0.05) and confirmed to be non-parametric in nature. The difference between simulation and control groups was thus subsequently evaluated using the Mann–Whitney U test (*p* < 0.05), with each component of the questionnaire being compared separately. Data analysis was carried out using Python, version 3.12.

## Results

In total, 47 medical students took part in the simulation and human factors module over one academic year. All students completed pre- and post-attachment questionnaires. In addition, 35 students (74 per cent) had prior simulation experience, predominantly from extracurricular society events.

The analysis of the 47 students’ pre- and post-attachment questionnaires revealed significant changes in their opinions and attitudes. Although the students did not demonstrate an increased interest in pursuing a career in surgery (*p* = 0.6) or otolaryngology (*p* = 0.17) following the rotation, they exhibited heightened comfort levels with the specialty, emphasising the positive impact of simulation-based learning (Table 1).

**Table 1.** Pre- and post-attachment questionnaire mean scores and their statistical analysis of intervention

Parameter	Pre-attachment questionnaire (*n* = 47)	Post-attachment questionnaire (*n* = 47)	*p* value
Interested in a career in surgery	2.71	3.01	0.0604
Interested in a career in otolaryngology	2.37	2.98	0.17
Self-assessed otolaryngology knowledge	2.09	3.88	<0.05
Self-assessed comfort levels in the operating theatre setting	3.30	4.22	<0.05
Importance of simulation training	3.74	4.68	<0.05
Importance of human factors training	3.76	4.63	<0.05

Additionally, the study identified a shift in students’ perceptions regarding the importance of specific qualities and skills for a surgeon, with technical skill and work ethic the most commonly cited attributes required to be a surgeon. Furthermore, improvements in comfort with otolaryngology knowledge (*p* < 0.005) and the operating theatre setting (*p* < 0.005) suggested the effectiveness of the educational interventions. At the conclusion of the module, students’ attitudes reflected their view on the importance of simulation (*p* < 0.05) and human factors (*p* < 0.05).

## Discussion

Simulation is fast becoming an integral part of medical education. Its benefits in enhancing learning and improving the educational experience are well established. Students who have undergone simulation training are more competent and confident junior doctors.^2^ They can experience real-world scenarios and learn from their mistakes. These aspects of simulation are frequently discussed in the literature, but a point that is often forgotten is that simulation allows students to experience the day-to-day components of various specialities. In a speciality such as otolaryngology, where students may get limited clinical exposure, this can be a salutary experience. Our simulation module allowed students to understand the clinical nuances of otolaryngology procedures and experience the satisfaction of a successful procedure. Coupled with our human factors discussions, our module equipped students with the tools required to become proficient junior doctors.

Whilst it has long been accepted that knowledge and technical skill are two of the key attributes required to be a surgeon, in recent years non-technical or soft skills are becoming more and more important.^7^ Effective communication, teamwork and professionalism are imperative in medical practice. Previously, these were thought to be inherent traits, but recent evidence has shown that they can be learned and improved through standardised educational programmes.^8^ More importantly, improving these skills enhances patient satisfaction and can improve patient outcomes.^9^

Medical educators have identified the shift to outcome-based learning, and entrustable professional activities have been utilised to assess competencies prior to progression into clinical practice.^6^ As such, our programme is an ideal platform to incorporate entrustable professional activities into Irish healthcare education. Key competencies are identified, then these are observed and evaluated by senior clinical staff. Both positive and negative feedback is imperative. Additionally, with the introduction of entrustable professional activities, the education experience for each student can be standardised.

- Simulation-based education enhances understanding and retention in medical training, but otolaryngology is often underrepresented in undergraduate curricula, limiting student exposure
- Non-technical skills (e.g. communication, professionalism and teamwork) are essential for modern surgical practice and are now emphasised in surgical training
- Entrustable professional activities are increasingly used to assess clinical readiness and competence
- This study introduced a novel two-week otolaryngology module integrating simulation, entrustable professional activities and human factor discussions at undergraduate level
- Significant improvements in students’ comfort with otolaryngology knowledge and operating theatre environments were demonstrated, and an enhanced appreciation for simulation and human factors in surgical training was observed
- Integrating technical and non-technical training can positively shift perceptions of essential surgical attributes, but may not directly increase interest in surgical careers

## Conclusion

This study highlighted the value of incorporating entrustable professional activities, lectures and discussions on human factors and innovative simulations in a two-week otolaryngology rotation for medical students. The observed changes in comfort levels and perceptions of essential skills underscore the positive impact of this comprehensive educational approach. The findings support the integration of simulation-based learning and human factor lectures and discussions to enhance surgical education, addressing both technical skills and the crucial non-technical aspects of healthcare. As medical education evolves, this study contributes valuable insights into optimising training paradigms for future surgeons, emphasising the holistic development of the skills and qualities necessary for a successful career in otolaryngology.

## Data Availability

The data that support the findings of this study are openly available in PubMed Medline, available at https://pubmed.ncbi.nlm.nih.gov.

## References

[1] Norman G. Medical education: past, present and future. *Perspect Med Educ*. 2012;1(1):6–14. 23316454 10.1007/s40037-012-0002-7 PMC3540368

[2] McInerney N, Nally D, Khan MF, Heneghan H, and Cahill RA. Performance effects of simulation training for medical students - a systematic review. *GMS J Med Educ*. 39(5):Doc51. 10.3205/zma001572 PMC9733478 36540561

[3] Ishak W, Nikravesh R, Lederer S, Perry R, Ogunyemi D, and Bernstein C. Burnout in medical students: A systematic review. *Clin Teach*. 2013;10(4):242–5. 23834570 10.1111/tct.12014

[4] Doherty E, O’Keeffe D, and Traynor O. Developing a human factors and patient safety programme at the Royal College of Surgeons in Ireland. *Surgeon*. 2011;9(Suppl 1). S38-9. 21549995 10.1016/j.surge.2010.11.003

[5] Leape L, Berwick D, Clancy C, Conway J, Gluck P, Guest J, et al. Transforming healthcare: a safety imperative. *Qual Saf Health Care* 2009;18(6):424–8. 19955451 10.1136/qshc.2009.036954

[6] Sanner Dixon K, Raheel A, Adkins S, Johnson BM, Ayres JM, Pruss O, Minchew HM, Riffel J, Berbel G, and Kilgore LJ. Holding the Knife on Perceptions of Surgery. *J Surg Educ*. 2024 Nov;81(11):1513–1521. 39217682 10.1016/j.jsurg.2024.07.031

[7] Woods MS, Liberman JN, Rui P, Wiggins E, White J, Ramshaw B, and Stulberg JJ. Association between Surgical Technical Skills and Clinical Outcomes: A Systematic Literature Review and Meta-Analysis. *JSLS*. 2023 Jan-Mar;27(1):e2022.00076. 10.4293/JSLS.2022.00076 PMC9913064 36818767

[8] Raper SE, Gupta M, Okusanya O, and Morris JB. Improving Communication Skills: A Course for Academic Medical Center Surgery Residents and Faculty. *J Surg Educ*. 2015 Nov-Dec;72(6):e202–11. 26183787 10.1016/j.jsurg.2015.06.008

[9] NHS England, 2019. Improving communication between healthcare professionals and patients in the NHS in England: Summary report. Available at: https://www.england.nhs.uk

